# Insights into the Interaction Between *Clostridioides difficile* and the Gut Microbiome

**DOI:** 10.3390/jpm15030094

**Published:** 2025-02-28

**Authors:** Dimitra Mougiou, Georgia Gioula, Lemonia Skoura, Cleo Anastassopoulou, Melania Kachrimanidou

**Affiliations:** 1 Department of Microbiology, Medical School, Aristotle University of Thessaloniki, 54124 Thessaloniki, Greece; dmougiou@auth.gr (D.M.); ggioula@auth.gr (G.G.); 2 Department of Microbiology, AHEPA University Hospital, 54124 Thessaloniki, Greece; lemskour@auth.gr; 3 Department of Microbiology, Medical School, National and Kapodistrian University of Athens, 11527 Athens, Greece; cleoa@med.uoa.gr

**Keywords:** *Clostridium difficile* infection, gut microbiota, gut microbiome, microbial diversity

## Abstract

*Clostridioides difficile* (*C. difficile*) is a significant healthcare-associated pathogen that is predominantly caused by antibiotic-induced microbiota disturbance. Antibiotics decrease microbial diversity, resulting in *C. difficile* colonization and infection. *Clostridium difficile* infection (CDI) manifests through toxins A and B, causing diarrhea and colitis. Antibiotic usage, old age, and hospitalization are significant risk factors. A healthy gut microbiota, which is dominated by *Firmicutes* and *Bacteroidetes*, provides colonization resistance to *C. difficile* due to competition for nutrients, creating inhibitory substances and stimulating the immune response. Antibiotic-induced dysbiosis decreases resistance, allowing *C. difficile* spores to transform into vegetative forms. Patients with CDI have decreased gut microbiota diversity, with a decrease in beneficial bacteria, including *Bacteroidetes*, *Prevotella*, and *Bifidobacterium*, and a rise in harmful bacteria like *Clostridioides* and *Lactobacillus*. This disparity worsens the infection’s symptoms and complicates therapy. Fecal Microbiota Transplantation (FMT) has emerged as a potential therapy for recurrent CDI by restoring gut microbiota diversity and function. Comprehending the connection between gut microbiota and CDI pathogenesis is critical for establishing effective preventive and treatment plans. Maintaining a healthy gut microbiota through careful antibiotic use and therapeutic options such as FMT can help in the management and prevention of CDI.

## 1. Introduction

*Clostridioides difficile* (also referred to as *Clostridium difficile*) is among the most significant pathogens linked to healthcare that disrupt the gut microbiota mostly by the extended use of antimicrobials. Exposure to antibiotics is the primary risk factor for CDI because it alters the gut microbiota’s normal composition and function. The intestinal flora goes into a state of dysbiosis, and the growth of *C. difficile* is favored [1,2].

Dysbiosis of the gut microbiota is involved in the pathogenesis of various diseases, and the case of CDI is the best-studied example. Patients with CDI have been found to have significant differences in their gut microbiota compared to healthy individuals. The two main populations of the gut microbiota, *Firmicutes* and *Bacteroidetes*, are reduced, and there is an increase in *Proteobacteria*, which include several harmful bacteria [3,4].

The administration of antibiotic agents is an important risk factor for the development of CDI. Many antibiotics reduce populations of bacteria that produce bile salt hydrolysis enzymes. As a result, the conversion of primary bile acids to secondary bile acids is suspended. Secondary bile acids inhibit the growth of *C. difficile*. Hence, their reduction creates a favorable environment for the growth of *C. difficile*. In fact, the ratio of primary to secondary bile acids is a predictive factor for the development of recurrent CDI [5].

*C. difficile* is found in two forms: the vegetative form and the spore form that is resistant to antibiotics and responsible for its transmission. The disease develops when the spores colonize the gut, develop into vegetative cells, and produce toxins such as toxin A (TcdA), toxin B (TcdB), and, less commonly, binary toxin (CDT). These toxins trigger several biological mechanisms that result in fluid secretion, inflammation, and tissue destruction, which in turn causes CDI symptoms like diarrhea and stomach pain. The development of CDI depends on disruptions in the gut microbiota, and recovery depends on restoring the diversity and abundance of homeostatic bacteria. Therefore, in recent decades, interest in the microbiome and in the microbiota has grown as a potentially promising area for the therapeutics of CDI [2].

Antibiotics are usually used to treat the first episode of CDI. The antibiotics mainly used are vancomycin and fidaxomicin. Oral administration of vancomycin leads to a reduction in *C. difficile* in the feces, with a consequent reduction in the likelihood of infection in other patients. However, like all antibiotics, it causes further destruction of the gut microbiota and further increases the risk of CDI. In addition, its administration is associated with an increase in vancomycin-resistant *Enterococci* in the gut [6,7]. Fidaxomicin has a therapeutic effect against *C. difficile* without harming the gut microbiome and tends to replace vancomycin in the therapy of CDI [8].

FMT is an alternative treatment for CDI, especially for recurrent CDI (rCDI), and has a high rate of 90%. Stools from healthy people are used in capsule form and the aim is to restore normal gut microbiota to reduce the likelihood of re-infection [9].

## 2. Clostridium Difficile Infection (CDI)

CDI is characterized as an important threat in the Antibiotic Resistance Threats Report presented by the American Center for Disease Prevention and Control (CDC). The CDC’s most recent surveillance data reports the crude overall incidence rate to be 116.1 cases per 100,000 persons, with a higher incidence of community-associated cases (62.1 cases per 100,000 persons) compared with healthcare-associated cases (54.0 cases per 100,000 persons) [10]. In addition, the onset of new ribotypes (RTs) through the last few years has increased the mortality and morbidity rates of CDI. The most frequent RTs are PCR RT027 and RT078. Their emergence has altered CDI epidemiology and the distribution of common RTs in various European countries [11].

*C. difficile* can be found in both urban and rural settings, while urban areas have greater pediatric patient incidence rates and notable antibiotic resistance. Numerous *C. difficile* strains, including extremely divergent and non-toxic strains, are also found in rural environments, especially soil. Both environments have potential for zoonotic transmission, which highlights the necessity of keeping an eye on and managing *C. difficile* in a variety of environmental sources [12,13,14].

The Gram-positive, spore-forming rod *C. difficile* is the cause of CDI, which can range from moderate diarrhea to potentially fatal pseudomembranous colitis. CDI is thought to be an infection associated with healthcare that is mainly spread by other CDI patients who also have symptoms.

The normal gut microbiota is the primary defense against CDI [15]. Transmission of *C. difficile* occurs through the spores of the microorganism by the fecal–oral route. The microorganism’s spores resist antimicrobials, heat, and disinfectants. Two toxins are produced by its toxigenic strains: toxin-A, which is an enterotoxin, and toxin-B, with cytotoxic activity. Both of these toxins act on the intestinal mucosa and destroy it, while B-toxin is a key factor in causing CDI [16]. Some *C. difficile* strains are classified as hypervirulent and cause severe disease. These strains produce large amounts of toxins A and B and also produce one other toxin, the binary toxin (tcdC), which has two components: CDTa and CDTb. These components cause the toxin’s action (CDTa) and its binding to the LSR receptor (lipolysis-stimulated lipoprotein receptor) (CDTb) [17]. The induction of CDI by these strains is not associated with prior antibiotic use or destruction of the gut microbiota [18].

*C. difficile* can be found both in the hospital environment and in the household environment. A key pillar in the management of CDI in the household environment is proper hand washing with soap and water before eating or after using the toilet. If an individual has diarrhea, then ideally, he should use a separate toilet from other people living in the same house, but if this is not possible, then the bathroom and all objects or parts of the house that come into contact with the patient should be cleaned with disinfectants that work against *C. difficile* spores. Based on the United States Environmental Protection Agency list, the main disinfectants recommended for household use against *C. difficile* spores are sodium hypochlorite, hydrogen peroxide, and hypochlorous acid. In addition, bed linen, towels and clothes of the patient should be washed with warm water and, if their composition allows, with a chlorine-based detergent [19,20].

Hospitalization, advanced age, and antibiotic exposure are important risk factors for the development of CDI [15]. Colonization by *C. difficile* can be prevented by the gut microbiota. Thus, colonization depends on several factors, such as the host, the microorganism, and the environment. The most common risk factors for *C. difficile* colonization are prior hospitalization, long-term antibiotic usage, proton pump inhibitor use, and immunosuppressive medication [21].

The use of antibiotics causes changes in the gut microbiota, such as a reduction in the diversity of the microorganisms that make it up [22]. Almost all antibiotics are associated with some degree of risk of CDI, including those used to treat the infection itself, such as metronidazole and vancomycin. CDI is closely linked to the use of cephalosporins, clindamycin, ampicillin, amoxicillin, penicillin, and fluoroquinolones, which significantly alter the normal gut microbiota [23].

## 3. Gut Microbiota in Healthy Adults

Compared to all of the cells in the human body, the number of bacteria that colonize the gastrointestinal system is extremely high (1:1), and their genetic material exhibits 100 times greater genetic variety than the human genome as a whole [24]. The large intestine’s lumen contains the majority of them. These microorganisms interact with the host in many complex ways, but this interaction is not yet fully understood. The members of the gut microbiota belong to all three classes of microorganisms: Archaea, Bacteria, and Eukaryotes.

**Figure 1:**
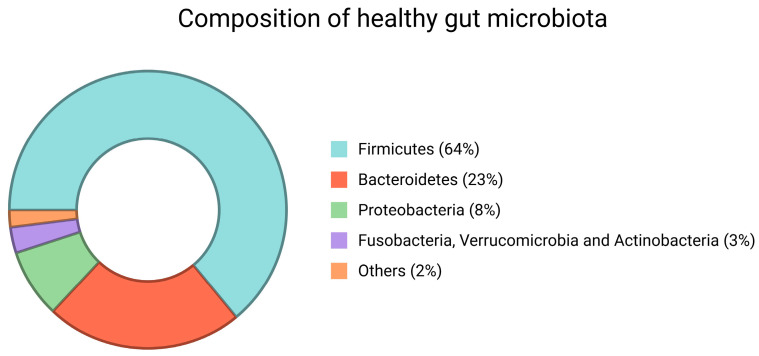
Composition of gut microbiota. *Firmicutes* and *Bacteroidetes* are the most represented phyla. *Proteobacteria*, *Fusobacteria*, *Verrucomicrobia*, and *Actinobacteria*. Created in BioRender.

Microbes from the mother and the surrounding environment colonize the newborn. As it grows, its gut microbiota acquires the structure of the adult’s, which is primarily composed of two categories of strictly anaerobic microbes from the phyla *Firmicutes* and *Bacteroidetes* [25]. These two phyla account for 90% of the bacteria detected in feces. The rest percentage belongs to *Proteobacteria*, which include Gram-negative bacteria (*E. coli* and *H. Pylori*), *Fusobacteria*, *Verrucomicrobia*, and *Actinobacteria*, which include genera such as *Bifidobacterium* [26] (Figure 1).

Gut microbes form biofilms on the intestinal mucus surface, which helps maintain homeostasis and protect against pathogens. A biofilm is a complex aggregation of microorganisms marked by the excretion of a protective and adhesive matrix [27,28]. Multiple bacterial, viral, fungal, and eukaryotic species form these biofilms, which are coated in an extracellular matrix made up of proteins, exopolysaccharides, extracellular DNA, and host and environmental elements [27]. Biofilms in the gut are dense and stable, and they contribute to nutrition exchange, colonization resistance, and as a barrier against infection [28,29].

Multiple types of interactions such as competition, neutralism, commensalism and mutualism are developed between the microbes of the gut community. Depending on the type of interactions between microbes, biofilms are created with different structure and characteristics. In neutralism, microbes use different nutrients without influencing one another, creating larger but isolated patches, but in competition, microbes compete for the same resources, resulting in segregated biofilm formations. In mutualism, microorganisms profit from one another’s metabolic by-products, creating highly intermixed and stable biofilm formations, while in commensalism, one bacterium benefits from the by-products of another, encouraging intermixing within the biofilm [28,29].

Environmental factors such as diet, infections and host factors, including immune responses play a crucial role in maintaining biofilm structure and function [27,28,29]. Nutrient absorption, immune system regulation, and pathogen defense are all facilitated by healthy biofilms. Diseases include inflammatory bowel disease (IBD), colorectal cancer (CRC), and infections by opportunistic pathogens that can result from disruption of biofilms. Novel treatments for microbiota dysbiosis may result from an understanding of biofilm dynamics and microbial interactions [27,28,29].

Over a person’s lifetime, a variety of factors, including their nutrition, environment, age and medication can impact the composition of their gut microbiota. As a result, it is considered that everyone has a unique gut microbiota, his or her own microbial fingerprint. However, there are specific features that distinguish the “healthy” from the “diseased” state, described in the form of specific patterns common among the symbiotic microorganisms that make up the gut microbiota [30,31]. Thus, shifts in some phyla’s abundances and/or a decrease in species diversity could be signs that a microbiome has strayed from a homeostatic or healthy condition.

## 4. Functions of Gut Microbiota

The microorganisms that make up the gut microbiota play an important role in health and disease [32]. Μicrobes in the gut microbiota produce substances called metabolites that affect both the host and other microbes in the intestinal tract. Short-chain fatty acids (SCFAs), indole, bile acids, amino acids, and LPS are the most frequently produced metabolites. Through metabolites gut microbiota’s bacteria exert many functions that are summarized in Table 1 [33,34].

For their production, gut microbiota uses the nutrients in the intestinal tract, modifies the metabolites produced by the host, or creates new ones from the beginning. Their chemical structure is like the chemical structure of the metabolites produced by the host, and their action on the host is similar [34].

## 5. The Role of Gut Microbiota in CDI Pathogenesis

The gut microbiota significantly influences the pathophysiology of CDI, through several types of procedures.

Colonization Resistance: Healthy gut microbiota ensures protection against colonization of the gut with pathogens like *C. difficile* by competing for nutrients, producing inhibitory substances, and stimulating the host’s immune response [35,36]. Disruption of this microbiota, often due to antibiotic use, reduces colonization resistance, allowing *C. difficile*’s spores to germinate and proliferate.

Between *C. difficile* and gut microbes, there is competition for nutrient elements, and this favors the growth of *C. difficile* [37]. In particular, *C. difficile* competes with *C. scindens* and *C. hiramonis* for the amino acid proline, with some strains of *C. difficile* showing an advantage in this competition because they have a mechanism for the positive regulation of proline reductase genes [37,38]. In addition, *C. difficile* can use mannitol as an alternative energy source, whereas *C. scindens* cannot. Thus, it has an advantage over other microbes in its ability to survive in environments with alternative nutrient substrates [37,38]. *C. difficile* has the ability to adapt to different nutritional environments, and this ability favors its growth especially in conditions where the gut microbiota is disturbed, as is seen after taking antibiotics [37,38].

**Figure 2:**
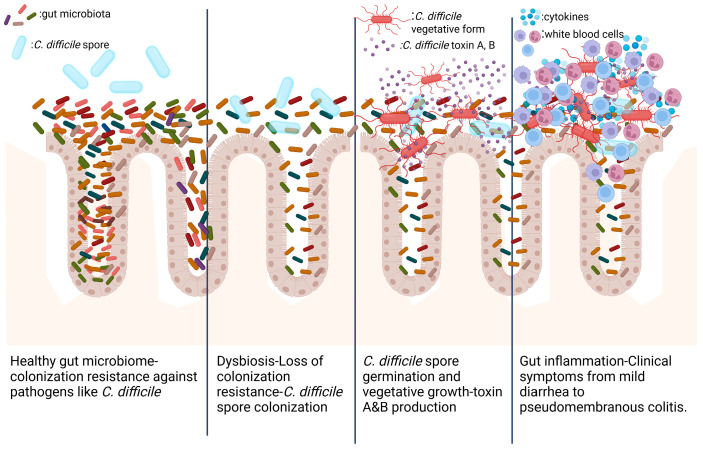
CDI pathogenesis. Created in BioRender.

Gut Microbiota Dysbiosis: Antibiotic usage is a significant risk factor for CDI as it disrupts the normal gut microbiota, reducing its diversity and altering its metabolic functions [39]. The state of dysbiosis in which the intestinal microbiota is involved makes the gut environment vulnerable to colonization of *C. difficile* and to the development of infection, as shown in Figure 2. Antibiotic treatment favors *C. difficile* colonization and infection by disrupting the microbiome, reducing protective bacteria and metabolites, altering bile acid metabolism, and modulating the immune response. Antibiotics of a broad spectrum can disrupt the normal gut microbiota, leading to a decreased overall diversity and specifically to a decrease in the abundance of Firmicutes and an increase in *Proteobacteria* [4]. They also eliminate bacteria that produce metabolites inhibitory to *C. difficile*, such as secondary bile acids and short-chain fatty acids (SCFAs). The loss of these protective bacteria and their metabolites can facilitate *C. difficile* spore germination and vegetative growth. Antibiotic induced alterations in the gut microbiome can impact the immunological response of the host. A weakened or altered immune response can reduce the ability to control *C. difficile*, leading to infection. It should be noted that prolonged antibiotic use might cause the gut microbiota to be repeatedly disrupted, which can result in recurrent CDI by allowing *C. difficile* spores to persist and re-germinate [35]. As mentioned above, all antibiotics are associated with some degree of risk of CDI. Although fluoroquinolones, cephalosporins, and clindamycin are linked to a higher risk for CDI [40].

### Gut Microbiota-Derived Metabolites

Secondary Bile Acids: The pathogenesis of CDI is significantly influenced by bile acids. The gut microbiota can be affected by antibiotic exposure, which can result in the loss of bacteria responsible for producing bile salt hydrolases (BSHs). By converting primary bile acids to secondary bile acids, these enzymes prevent *C. difficile* from proliferating and reduce the activity of its toxins. The gut becomes rich in primary bile acids when BSH-producing bacteria are reduced, which can cause *C. difficile* spores to germinate leading to infection [41,42].

Short-Chain Fatty Acids (SCFAs): By strengthening the intestinal barrier, modulating the immune response and inhibiting *C. difficile*, SCFAs contribute to gut health [43]. SCFAs consist of saturated organic acids with one to six carbons. The most prevalent are acetate, propionate and butyrate. Dysbiosis leads to a decrease in SCFA production, weakening the gut’s defense against *C. difficile*.

Specific Gut Microbiota Signatures: Certain bacterial families and genera are associated with either resistance or susceptibility to CDI [40]. For example, a reduction in beneficial bacteria such as *Lachnospiraceae* and *Ruminococcaceae* and an increase in harmful bacteria like *Enterobacteriaceae* are linked to CDI pathogenesis [44,45].

Age and Other Factors: Advanced age, hospitalization, and the use of proton pump inhibitors (PPIs) also impact gut microbiota composition, further increasing the risk of CDI. As the age advances, the risk of developing CDI increases [46,47,48]. Gut microbiota loses its stability, and its structure changes. More precisely, *Bifidobacterium* and *Firmicutes* decrease while *Bacteroidetes* and *Proteobacteria* increase [48,49,50]. The use of proton pump inhibitors (PPIs) is related to CDI, particularly in combination with antibiotics [51]. It has been shown in vitro that PPIs have an impact on the development of *Lactobacillus* [52] and simultaneously cause a reduction in *Bacteroidetes* and a rise in *Firmicutes* at the phylum level, an increase in *Holdomaniafiliformis* at the species level and a decrease in *Pseudoflavonifractorcapillosus* [53]. Prolonged hospitalization is also a significant risk factor for CDI onset. Several studies demonstrate that after one year of hospitalization colonization is possible in up to 50% of patients [54,55]. The main cause is the transfer of *C. difficile* spores from various surfaces to patients via staff. Patients cared for at home have a lower risk (15%) than hospitalized patients [15] but higher than the general population.

Fecal Microbiota Transplantation (FMT): Recurrent CDI can be effectively treated with FMT, which aims to restore the diversity and function of the gut microbiota. It replenishes the gut microbiota, re-establishing colonization resistance and improving gut health. Fecal transplantation is a technique known since ancient times. In modern times, it was first used in 1958 by Eisenman et al. for the treatment of pseudomembranous colitis, the cause of which was not known at the time [56,57]. Samples of stool from a healthy donor are introduced via the mouth (capsules) or the rectum (colonoscopy). The combination of FMT together with discontinuation of antibiotics is the most successful therapeutic approach in relapsed CDI, with a success rate of 75–90% [15,16]. Patients where this therapy was applied showed higher recovery rates (90%) compared to those on antibiotic treatment (60%). In addition, the increase in *Bacteroidetes* and non-pathogenic *Clostridia* observed after fecal transplantation led to suppression of *C. difficile* growth [33]. The possibility of infectious microorganisms being transferred from the donor to the receiver and the emergence of autoimmune diseases are the two main causes of concern. Appropriate donor screening reduces the risk of fecal material infection while it remains possible. Concerns over the negative effects of fecal transplantation that can appear after a long time are raised by the impact of gut microbiota on certain immune-related disorders, such as irritable bowel disease. This should be further investigated in the future [15].

## 6. The Effect of CDI on the Diversity of the Gut Microbiome

CDI significantly affects gut microbiome diversity. A gut microbiota full of *Bacteroidetes* and SCFA-producing bacteria confers colonization resistance against *C. difficile* by maintaining a balanced metabolic environment, including amino acid and fatty acid metabolism. Disruption of this balance leads to reduced microbiota diversity and a decrease in beneficial bacteria, which impacts the metabolism of fatty and bile acids. This disruption creates an environment that is conducive to *C. difficile* proliferation [58].

**Figure 3:**
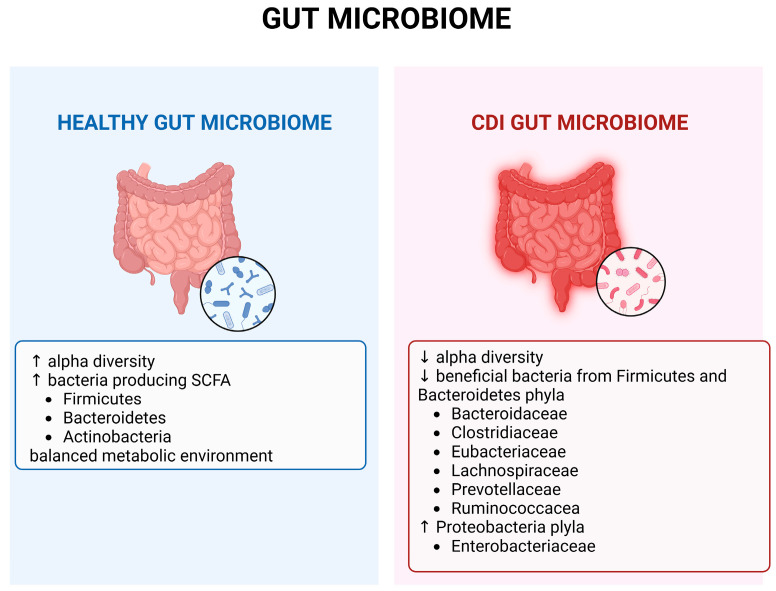
Gut microbiome in health and in CDI. Created in BioRender.

Patients with CDI show significant differences in microbiome diversity compared to healthy individuals [4]. In CDI patients, the gut microbiome has low diversity and less beneficial bacteria such as *Bacteroides*, *Prevotella*, and *Bifidobacterium* species while displaying a higher level of abundance of *Clostridioides* and *Lactobacillus* species (Figure 3). This imbalance allows *C. difficile* to thrive and produce toxins that cause colonic inflammation and clinical symptoms ranging from mild diarrhea to severe pseudomembranous colitis and toxic megacolon [59,60,61]. As for asymptomatic carriers of *C. difficile*, less is known about the composition of their gut microbiota. It is found that they have a smaller number of *Proteobacteria* and more *Firmicutes* and *Bacteroidetes* in their gut microbiota compared to patients with CDI. The composition of their gut microbiota is closer to that of a healthy person [62].

CDI patients exhibit lower richness and alpha diversity in their gut microbiome. This means there are fewer different species present and a less even distribution of these species compared to healthy individuals. Significant differences between individuals (beta diversity) are also observed between CDI patients and other groups, indicating distinct microbial community structures in those with CDI. In CDI patients, the most prevalent phylum is *Bacteroidetes*, followed by *Firmicutes*, which is the opposite of what is observed in healthy individuals where *Firmicutes* is more dominant. This shift in phylum dominance reflects the overall disruption in the gut microbiome [4].

## 7. Discussion

The diversity of the gut microbiota is significantly disrupted by CDI. Patients with CDI exhibit lower richness and alpha diversity, meaning there are fewer different species present and a less even distribution of these species compared to healthy individuals. This disruption leads to a decrease in beneficial bacteria such as *Bacteroides*, *Prevotella*, and *Bifidobacterium*, whereas increasing the quantity of harmful bacteria like *Clostridioides* and *Lactobacillus*. The imbalance favors *C. difficile* proliferation, exacerbating infection symptoms and complicating treatment and recovery. Additionally, CDI patients often show an elevated presence of opportunistic pathogens and a reduction in bacteria that produce SCFAs, further weakening gut health [4,58,63].

A study conducted by Vázquez-Cuesta et al. pointed to a notable decrease in beneficial bacteria such as *Bifidobacterium*, *Collinsella*, *Blautia*, *Butyrivibrio*, *Clostridium_XlVb*, *Coprococcus*, *Dorea*, *Fusicatenibacter*, *Ruminococcus*, and *Faecalibacterium* in the gut microbiome of CDI patients. These bacteria are important for maintaining gut health and preventing colonization by pathogens. Moreover, there was an increase in potentially pathogenic genera such as *Staphylococcus*, *Enterococcus*, *Lactobacillus*, *Streptococcus*, *Parvimonas*, *Clostridium_XlVa*, *Robinsoniella*, *Peptostreptococcus*, *Clostridium_XVIII*, *Coprobacillus*, *Veillonella*, *Fusobacterium*, *Campylobacter*, *Proteus*, *Pseudomonas*, and *Akkermansia*. These changes can exacerbate the infection and contribute to its symptoms [4].

Herrera et al. also demonstrated that CDI-negative patients maintain a more balanced and diverse microbial community with a predominance of *Firmicutes* and *Bacteroidetes* and a lower relative abundance of *Proteobacteria* and *Verrucomicrobia* whereas CDI-positive patients exhibit a disrupted and less diverse gut microbiota with increased opportunistic pathogens from the phyla of *Proteobacteria* and *Verrucomicrobia* [3]. The fecal sample was further tested for eukaryotes and Archaea, and it was found that CDI-positive patients had an increased abundance of fungi, particularly *Candida species*, a significant increase in microorganisms that belong to the phylum *Opalozoa* and a higher amount of various Archaea, except for *Methanobrevibacter*. Moreover, microbial interactions between bacteria and eukaryotes are more stable in CDI-negative patients than in CDI-positive [3].

Rea et al. made a comparison between the microbiota of healthy individuals and that of *C. difficile* carriers and patients with CDI. It was found that the microbiota of healthy subjects did not differ from that of *C. difficile* carriers when it comes to phyla and family level. However, a significant difference was observed in CDI patients’ microbiota compared to the two other groups. Patients diagnosed with CDI showed a reduction in microbial diversity at the genus level in comparison to those who did not develop *C. difficile* in their culture. Significant differences were also observed at the family level. Families such as *Erysipelotrichaceae*, *Aerococcaceae*, and *Flavobacteriaceae* were elevated in CDI, while *Enterococcaceae*, *Leuconostocaceae*, *Prevotellaceae*, and *Spirochaetaceae* were reduced. Moreover, there was an absence of *Bifidobacteria spp.* among patients with a history or present CDI, whereas they were present in non-symptomatic carriers and the group with negative *C. difficile* culture [64].

Additionally, patients with CDI and patients with diarrhea not related to *C. difficile* were compared to healthy individuals. In patients with diarrhea, no matter the pathogen, gut microbiota had less variety and reduced *Firmicutes* population. Microbiota of the control group was rich in *Bacteroidetes* species, *Lachnospiraceae*, and *Ruminococcaceae*, whereas the microbiota of individuals with diarrhea consisted of *Enterobacteriaceae*, *Erysipelotrichaceae*, and *Lachnospiraceae*. *Lachnospiraceae* and *Ruminococcaceae* produce butyrate, which inhibits *C. difficile* development, suppresses inflammation, and preserves colon health [65].

Herrera et al. demonstrated that CDI is associated with a decrease in beneficial bacterial genera like *Faecalibacterium*, *Dorea*, and *Lachnospira* and a rise in pathogens from the phylum *Pseudomonadota*, as well as fungi like *Candida*, *Malassezia*, and *Blastocystis*. This imbalance creates an environment conducive to *C. difficile* growth because there is a lack of bacteria that produce SCFAs and secondary bile acids, which are important for maintaining gut health [66].

The infection can alter the structure of the gut microbiome, favoring the growth of certain bacterial populations over others. For example, populations of pathogenic bacteria like *Enterococcus*, *Helicobacter*, and *Klebsiella* may increase, while beneficial bacteria associated with fiber degradation and bile acid metabolism, such as *Anaerotignum*, *Blautia*, *Lactonifactor*, and *Monoglobus*, may decrease [38].

Nzabarushimana et al. found that CDI patients exhibit a different microbial species/function profile compared to healthy individuals or those taking antibiotics. Specifically, CDI causes a decrease in microbial diversity and disrupts the balance of the gut microbiome, favoring the growth and proliferation of *C. difficile*. This disruption can result in a less diverse and less stable gut microbiome, making the gastrointestinal tract more susceptible to further infection and complications. Additionally, the use of antibiotics, which is common in CDI treatment, can further reduce gut microbiome diversity, exacerbating the dysbiosis associated with CDI [63].

In Table 2, we summarize the alterations of the gut microbiome due to CDI in the studies mentioned above.

## 8. Conclusions

Gut microbiota influences CDI pathogenesis by providing colonization resistance, producing inhibitory metabolites, and maintaining gut health. Disruptions in gut microbiota, often due to antibiotics, age, or other factors, lead to dysbiosis, reducing colonization resistance and increasing susceptibility to CDI. Understanding these mechanisms is crucial for developing effective prevention and treatment strategies for CDI [35].

The diversity of the gut microbiome is severely disrupted by CDI. Patients with CDI exhibit decreased richness and alpha diversity. This disturbance causes a decrease in beneficial bacteria and an increase in the percentage of harmful bacteria. The imbalance promotes *C. difficile* proliferation, causing exacerbation of the infection and complicating therapy and recovery [4,63].

Fecal Microbiota Transplantation (FMT) has emerged as an effective treatment for recurrent CDI, restoring gut microbiota diversity and function. Finally, understanding the interplay between gut microbiota and CDI pathogenesis is essential for developing effective prevention and treatment strategies [16].

## Figures and Tables

**Table 1:** Gut microbiota functions.

Gut Microbiota Functions	Gut Microbiota Metabolites
Metabolism of nutrients, xenobiotics and drugs	SCFAs, bile acids, Vitamin K and B complex, amino acids, indole, tryptophane
Integrity of gut barrier and gut motility	SCFAs, indole, LPS, bile acids, methane
Immunomodulation	SCFAs, bile acids, TMAO, LPS
Resistance to colonization	SCFAs, bacteriocins
Influence of the nervous system	LPS, SCFAs, GABA, BCAA’s
Modulation of the circadian rhythm	Bile acids, SCFAs

**Table 2:** Alterations of gut microbiome due to CDI.

Study	Gut Microbiome Changes Due to CDI	
	Increase	Reduction
Vázquez-Cuesta et al. [4]	*Staphylococcus*, *Enterococcus*, *Lactobacillus*, *Streptococcus*, *Parvimonas*, *Clostridium_XlVa*, *Robinsoniella*, *Peptostreptococcus*, *Clostridium_XVIII*, *Coprobacillus*, *Veillonella*, *Fusobacterium*, *Campylobacter*, *Proteus*, *Pseudomonas*, and *Akkermansia*.	*Bifidobacterium*, *Collinsella*, *Blautia*, *Butyrivibrio*, *Clostridium_XlVb*, *Coprococcus*, *Dorea*, *Fusicatenibacter*, *Ruminococcus*, and *Faecalibacterium*.
Herrera et al. [3]	*Proteobacteria*, *Verrucomicrobia*, *Candida species*, *Opalozoa* and various Archaea, except for *Methanobrevibacter*	*Firmicutes* and *Bacteroidetes*
Rea et al. [64]	*Erysipelotrichaceae*, *Aerococcaceae*, and *Flavobacteriaceae*	*Enterococcaceae*, *Leuconostocaceae*, *Prevotellaceae*, and *Spirochaetaceae*
Schubert et al. [65]	*Enterobacteriaceae*, *Erysipelotrichaceae* and *Lachnospiraceae*	
Herrera et al. [66]	*Pseudomonadota*, as well as fungi like *Candida*, *Malassezia*, and *Blastocystis*	*Faecalibacterium*, *Dorea*, and *Lachnospira.*
Lesniak et al. [38]	*Enterococcus*, *Helicobacter*, and *Klebsiella*	*Anaerotignum*, *Blautia*, *Lactonifactor*, and *Monoglobus*
Nzabarushimana et al. [63]	*Faecalibacteriumprausnitzii*, *Bacteroides thetaiotaomicron*, *Klebsiella pneumoniae*, *Prevotellacopri*, and *Ruminococcusgnavus*	

## Data Availability

No new data were created or analyzed in this study. Data sharing is not applicable to this article.
